# Effects of high volume saline enemas vs no enema during labour – The N-Ma Randomised Controlled Trial [ISRCTN43153145]

**DOI:** 10.1186/1471-2393-6-8

**Published:** 2006-03-19

**Authors:** Luis Gabriel Cuervo, María del Pilar Bernal, Natalia Mendoza

**Affiliations:** 1 Universidad Javeriana, Clinical Epidemiology Research and Training Center, Colombia; 2 Portland, OR, USA; 3 División de Investigaciones, Universidad El Bosque, Bogotá D.C., Colombia

## Abstract

**Background:**

Enemas are used during labour in obstetric settings with the belief that they reduce puerperal and neonatal infections, shorten labour duration, and make delivery cleaner for attending personnel. However, a systematic review of the literature found insufficient evidence to support the use of enemas. The objective of this RCT was to address an identified knowledge gap by determining the effect of routine enemas used during the first stage of labour on puerperal and neonatal infection rates.

**Methods:**

**Design: **RCT (randomised controlled trial; randomized clinical trial).

**Outcomes: **Clinical diagnosis of maternal or neonatal infections, labour duration, delivery types, episiotomy rates, and prescription of antibiotics

**Setting: **Tertiary care referral hospital at the Javeriana University (Bogotá, Colombia) that attended 3170 births during study period with a caesarean section rate of 26%.

**Participants: **443 women admitted for delivery to the obstetrics service (February 1997 to February 1998) and followed for a month after delivery. Inclusion criteria were women with: low risk pregnancy and expected to remain in Bogotá during follow up; gestational age ≥ 36 weeks; no pelvic or systemic bacterial infection; intact membranes; cervix dilatation ≤7 cm.

**Intervention: **1 litre saline enema, versus no enema, allocated following a block random allocation sequence and using sealed opaque envelopes.

**Results:**

Allocation provided balanced groups and 86% of the participants were followed up for one month. The overall infection rate for newborns was 21%, and 18% for women. We found no significant differences in puerperal or neonatal infection rates (Puerperal infection: 41/190 [22%] with enema *v *26/182 [14%] without enema; RR 0.66 CI 95%: 0.43 to 1.03; neonatal infection 38/191 [20%] with enema *v *40/179 [22%] without enema; RR 1.12, 95% CI 95% 0.76 to 1.66), and median labour time was similar between groups (515 min. with enema *v *585 min. without enema; P = 0.24). Enemas didn't significantly change episiorraphy dehiscence rates (21/182 [12%] with enema *v *32/190 [17%] without enema; P = 0.30).

**Conclusion:**

This RCT found no evidence to support routine use of enemas during labour. Although these results cannot rule out a small clinical effect, it seems unlikely that enemas will improve maternal and neonatal outcomes and provide an overall benefit.

## Background

Enemas are frequently used in obstetric settings because they are thought to reduce the risk of puerperal and neonatal infections, shorten the duration of labour and make delivery cleaner for attending personnel [1,2]. However, the use of enemas is controversial and there is little evidence of their effectiveness. Enemas are upsetting and humiliating for women in labour and increase the workload in labour wards. Enemas cause watery stools and could theoretically increase contamination and infection rates[3,4].

A systematic review of the literature found one trial with 222 women, which found no difference in puerperal or neonatal infections. However, it only followed women while they remained hospitalized; this time may be too short to identify outcomes that could be affected by enemas. The review concluded that there was insufficient evidence to recommend the use of enemas during labour and called for additional RCTs [5].

The main objective of this RCT was to find out if the use of high volume enemas during the first stage of labour modified neonatal and puerperal infectious rates. The null hypothesis stated that proportion of infections was similar for the intervention and control groups. The secondary objectives were to establish if there was an effect on specific neonatal or puerperal infectious rates, and other clinically relevant outcomes such as neonatal infections, maternal pelvic inflammatory disease or suture dehiscence, and antibiotic prescriptions. The protocol for this study was presented and debated at two meetings of the Latin American Clinical Epidemiology Network: Latinclen I in April 1995 in Colombia; and the Latinclen III in September 1997 in Dominican Republic.

## Methods

The RCT was conducted at the obstetric service of the *Hospital Universitario San Ignacio*, a tertiary care referral centre in Bogota, Colombia. Women attending the admission unit of the obstetrics' clinic for delivery were invited to participate.

In accordance with the protocol, the examining intern or resident would invite all eligible women to participate in the study. Recruitment rates varied greatly among different interns and residents. Because of the frequent rotation of interns and residents through the admission unit, interns and residents attended standardised monthly training sessions on how to enrol patients on the study.

Inclusion criteria included: absence of life threatening events at admission interview (such as placental abruptio, prolapsed cord, or eclampsia); gestational age ≥36 weeks based on the best available estimation such as a reliable last menstrual period date or an appropriate ultrasound; projected permanence in Bogota during the month following delivery; and willingness to participate expressed in a written informed consent. There was no age restriction for participants. However, for women under 18 years of age, an adult witness – most frequently a next of kin, was also asked to participate in the informed consent process.

Exclusion criteria included: a clinical diagnosis of any systemic or gynaecological bacterial infection; use of systemic antibiotics during the week prior to admission; rupture of amniotic membranes or uncertainty of their integrity; or a cervical dilatation >7 cm.

The trial protocol was approved by the Institutional Review Board of the Medical School of the Javeriana University and by the staff of the Clinical Epidemiology and Biostatistics Unit.

Women willing to participate were offered a range of additional services during follow-up. These services were designed to improve outcome detection and adherence to the study. All women received a booklet that addressed questions frequently raised by women during labour and puerperium (30 days following delivery), and informed on early signs for maternal and neonatal infections[6]. The development of the booklet involved semi-structured interviews carried out by a trained nurse who would elicit the issues that worried women during the first day, first week and first month after delivery. The team that designed the booklet included graphic designers and a social communications specialist. The booklet was tested and printed prior to the beginning of the RCT. Participants were offered two programmed health care visits where their concerns could be discussed with a professional nurse. During the visits, the nurse examined the mother and the baby and assessed outcomes. Systematic telephone reminders for these visits were scheduled before the 1^st ^and 4^th ^week after delivery. Women received instructions on how to access a 24-hour paging service which allowed them to contact a health care provider to address any concerns, seek support, inform about problems, or get advice on issues such as emergency medical attention. This allowed retrieving relevant information of visits to other healthcare providers and contacting them to collect data. The local branch of La Leche League International, a volunteer organization delivering support and education on breastfeeding, offered regular free educational sessions for participants. All participants were offered subsidised screening for neonatal hypothyroidism; when this RCT was done, screening for thyroid disease was not mandatory nor covered by health maintenance organisations in Colombia.

### Assignment and follow-up procedures

Randomisation was done in blocks of 2 (20%), 4 (60%) and 6 (20%) using Ralloc^® ^allocation software. Once the participant had completed the informed consent process, an opaque envelope with sequential numbering and instructions was opened. Women randomised to the intervention group received a 1 litre Travad^® ^2.5% sodium chloride solution enemas applied by a nurse assistant prior to been taken to the labour wards, where participants in both groups would thereafter receive the same care.

During the first and third week after delivery, reminders to attend scheduled appointments at the outpatient primary care clinic were delivered by phone and mail. We used a standardised telephone survey to assess if participants had used any other health services or been diagnosed with any particular condition. Newborns were screened for hypothyroidism during the first scheduled visit when the mother agreed to it. Results for the screening tests were scheduled to be delivered at the second visit, but these were also couriered with an explanatory note if the appointment was missed. The screening scheme covered confirmation diagnostic tests, when necessary.

The primary outcomes were the diagnosis of infections in newborns or women during the month following delivery. A neonatal outcome was positive if during the first month of life the child was prescribed systemic antibiotics. A neonatal outcome was also positive when the child was diagnosed with any of the following clinical conditions: ocular infection (purulent drainage in the eye after the sixth day of delivery), umbilical infection (foul smell with periumbilical erythema), skin infection (cellulitis or impetigo), lower or upper respiratory tract infection, intestinal infection, meningitis or sepsis.

A puerperal outcome was positive when, during the first month after delivery, a health care provider diagnosed the women with any of the following: dehiscence of the episiorraphy suture, purulent effusion from the episiorraphy, urinary tract infection, pelvic inflammatory disease, or vulvovaginitis.

The primary outcome of the study was an aggregated maternal and neonatal infection rate: either the mother or the newborn had an infectious outcome (combined infection rate) .

A team member visited participating women and newborns in hospital on a daily basis. Throughout the trial, trained research assistants using standardised questionnaires, registered data from telephone calls, hospitalisations, follow-up visits, and any communications with the participants, their families, or their health care providers.

### Masking

Masking the use of enemas was unfeasible. However, we made efforts to conceal the intervention by not separating documents with information on the allocation from those outcome data collection, by training the team's supporting clinical team (professional nurse, family medicine residents, family medicine staff, and consulting dermatologist) to avoid enquiring in ways that would unmask the allocation. Health care providers in other settings, such as physicians at emergency wards, paediatricians and medics at outpatient clinics were unaware of the allocation and frequently of the specific objectives of the study. Except for the intervention, participants received the same health care, and data retrievers would remain unaware of individual allocations. Interventions remained coded for the analysis and the code was broken once the analysis was completed. Input between data at recruitment and allocation was done weeks before the collection of data on outcomes at follow up.

### Sample size determination

We were unable to find reliable data on the incidence of baseline infection rates for puerperal women or newborns so we did a pilot study to have base data that would allow a good estimate of frequencies for sample size calculations. The pilot study, which included the first 44 participants of the control group, estimated the combined infection rate of puerperal women and newborns at 46 percent [7]. Using 5% significance and a 80% power, a sample of 394 participants distributed in two parallel groups was estimated to be required to detect a relative difference of 25% in combined infection rates of women and newborns, following the formulas provided by Duppont and Plummer[8]. Assuming 4% of the participants would be lost to follow-up, an estimated total sample of 410 women was required.

### Data management and analysis

The database created in Epi-Info v 6.04 b was fed using double data entry and transferred to Stata 5.0^© ^using Stata Transfer 4.0^©^. A Shapiro-Wilk test was used to determine if the distribution of continuous variables was normal. Non-normal distributions were transformed using a log transformation, and if the distribution was persistently non-normal, a Mann-Whitney test was used to compare groups. Bivariate analyses were done using the chi-square test or Fisher's exact test. Power calculations were done using specialized software developed at the Clinical Epidemiology Unit at the Javeriana University[9].

## Results

During the twelve months recruitment period (Feb 1997–Feb 1998) 3170 women were admitted for delivery to the obstetric service. The caesarean section rate in the obstetrics service was 26% at the time.

Of the 460 women interviewed for recruitment, 16 were non-eligible and 1 declined to participate. We randomised 443 women (see **Figure 1**), among which we had 12 protocol violations; 4 in the enema group and 8 in the control group. Nevertheless, these women were offered the care and benefits that all other participants had. Protocol violations included admission with ruptured amniotic membranes (5 women in the control group), infection at admission (1 in the control group, 2 in the enema group). Five women didn't fulfil inclusion criteria but were randomised (2 in the control group and 3 in the enema group). The analysis for the remaining women was done by group of allocation. Women who delivered by caesarean section were considered in the analysis. Data were not available to include in an 'intention to treat' analysis the 12 women excluded because of violations to the selection criteria (protocol violations).

Follow up was completed by 87% of the participating women and 86% of newborns. Direct examination by a team member at one month follow up was carried out in 20% of women and 19% of newborns (*P *= 0.51 and *P *= 0.98 respectively, with similar distribution between groups); standardised telephone interviews with participants and healthcare providers allowed to assess outcomes from the remaining participants.

Baseline characteristics were similar in both groups, suggesting that randomisation provided well-balanced and comparable groups **(Table 1 **[see additional file 2]**)**. Labour duration times and other maternal outcomes were obtained from women's records after delivery and are presented in **Table 2 **[see Additional file 2]. Neonatal baseline data obtained shortly after delivery from newborns' records are summarised in **Table 3 **[see Additional file 2].

We found no statistically significant differences between groups for labour duration, delivery types, episiotomy rates, or prescription of antibiotics. Caesarean sections were done in 12% of women with no significant differences in rates between groups.

No significant differences were found in the distribution between groups for newborns' "Ballard" score, birth weight, diagnosis of neonatal apnoea, or the administration of ocular and umbilical prophylaxis. Five newborns allocated to the control group and none in the treatment group developed respiratory tract infections, but this difference had no statistical significance. Two out of the five newborns who developed lower respiratory tract infections were delivered by caesarean section. The three newborns with omphalitis belonged to the intervention group, but again this difference was not statistically significant. Similarly, no significant differences were found for ophthalmic infection rates, skin infections, intestinal infections or the need for systemic antibiotics (**Table 4 **[see Additional file 2]).

No statistically significant differences were found for any of the assessed outcomes in puerperal women. Pelvic infections affected 4% of women: one was diagnosed with myometrytis; five with endometritis; three had vulvovaginitis; and six had infected episiorraphy sutures. The frequency and severity of perineal tear was similar in the intervention and control group. No significant differences were found in the rates of suture dehiscence among the 372 women who had epysiorraphy **(Table 5 **[see Additional file 2]**)**.

Breast pain complaints were not categorised as an infectious outcome and affected 31% of women, with 79% of them suffering breast engorgement or nipple cracking and no significant difference between groups (*P *= 0.75).

Summarised outcomes are provided in **Table 6 **[see Additional file 2]. Overall, one in five newborns had an infectious outcome, and rates were not statistically significant between groups. No significant differences were found for aggregated maternal infections, although the study may have been underpowered to rule out such differences; We found an 8% absolute risk difference and a broad, skewed confidence interval (see **Table 6 **[see Additional file 2]).

The aggregated outcome of "neonatal or puerperal infection" during the 30-day follow-up was higher in the control group than in the intervention group. However, the 6% difference in absolute risk can be due to chance (*P *= 0.23). Infections affecting both child and mother in the same family were not significantly different (6/183 [3%] with enema *v *5/191 [3%] with no enema; *P *= 0.39).

We planned to assess the effect of enemas' on labour duration using multiple linear regression to adjust for parity. However the normality test of the variable was rejected and a Boxcox transformation (with a range between -2 and 2) did not provide an appropriate model. The residuals analysed through a robust regression had a non-normal distribution, so a non-parametric quintile regression was done, finding no significant differences of labour duration within study groups after adjusting for parity (*P *= 0.07).

### Participant flow and follow-up

Described in **Figure 1 **[see Additional file 1].

## Discussion

Puerperal and neonatal infections, although seldom life threatening, were very frequent in this study. Ophthalmic infections were the most frequent infections amongst newborns. Breast engorgement and nipple cracking were the most frequent maternal complaints during puerperium. Episiorraphy dehiscence was the most frequent infectious outcome in women. We were impressed by how frequent these outcomes were, and it is likely that these problems are being missed in studies with a shorter follow up, such as those that only follow women during hospitalization. It also suggests that the follow up strategy probably had a good sensitivity.

High volume enemas used during the first stage of labour did not have a significant effect in the incidence of puerperal or neonatal infections, or labour duration. This RCT found no significant differences in puerperal or neonatal infection rates with enemas. No statistically significant effects were found when analysing women or newborns separately or when their outcomes were aggregated to analyse them as mother-newborn dyads.

The RCT had a power in excess of 80% to find differences as big as 25% in the aggregated infection rates for women and newborns. However, the study may have been underpowered to detect differences for individual outcomes. This RCT had an estimated power of 56% to find differences in neonatal infections, and 61% power to detect puerperal infections. A higher rate of operative vaginal deliveries was found in the enema group, although it did not reach statistical significance. It is worth mentioning this, because operative vaginal deliveries may have an effect in puerperal infection rates. The use of an aggregated outcome helped to reduce sample size but it would have been ideal to have a sample size large enough to establish effects in newborns and puerperal women separately. Despite being practical, aggregating results has important limitations: if the maternal and neonatal outcomes have significantly different magnitudes or point out in different directions, aggregation will cancelled out or underestimate the differences.

We didn't have resources to collect information to assess if the population of women admitted to the trial represented all eligible women. However, participation in the trial was apparently determined by the commitment of recruiters, not the participants' risk.

The RCT did not evaluate women's preferences or known adverse effects of enemas, such as pain, discomfort, embarrassment, or diarrhoea.

Since just one-fifth of the participants were personally examined by trained research assistants at the one-month assessment, measurements of these outcomes may have been imprecise and could potentially disguise existing differences, accounting for the lack of differences (risk of Type II error). Nevertheless, significant misrepresentations of outcomes grave enough as to require hospitalisation, dedicated care, or urgent consultations are unlikely with the follow up strategy we endorsed. Overall, participants and their families were helpful and willing to provide information; and we went to great efforts to use more than one information source and verify abnormal results in both women and neonates.

## Conclusion

Puerperal and neonatal infections had high incidence rates. Severe infections, such as myometritis and omphaylitis, were unusual. In this study enemas didn't significantly modify puerperal or neonatal infection rates. A dramatically larger study would be necessary to determine the effects, if any, of enemas on specific outcomes including life-threatening complications. Enemas cause discomfort, increase workload for health carers and marginally increase the cost of health care. At this time there is no good evidence supporting the routine use of enemas. It seems unlikely that the effect of enemas on the incidence of specific outcomes is large enough to outweigh the inconvenience or adverse effects associated to the routine use of enemas during labour.

The data from this study combined from additional RCTs may help better understand the particular effects of enemas, guide policies and elucidate the role of enemas during labour.

## Competing interests

The author(s) declare that they have no competing interests.

## Authors' contributions

**Luis Gabriel Cuervo **led the conception and design of the RCT, coordinated its implementation and development, data recollection, analysis and interpretation of data, and leads the writing of this manuscript.

**María del Pilar Bernal **trained research assistants, collected and input data, helped with the day to day management, participated in the writing of this article, and has reviewed its different versions.

**Natalia Mendoza **contributed to the development of data capturing templates, trained research assistants, collected and input data, and participated in the original writing of this article and has reviewed its different versions.

## Pre-publication history

The pre-publication history for this paper can be accessed here:



## Supplementary Material

Additional File 1 Participant flow and follow-up. Click here for file

Additional File 2 Characteristics of participants at admission. Maternal postpartum outcomes. Neonatal baseline data. Neonatal infectious outcomes. Maternal infectious outcomes. Grouped infectious outcomes. Click here for file
