# Supplementary figures and images for: Effects of high volume saline enemas vs no enema during labour – The N-Ma Randomised Controlled Trial [ISRCTN43153145]
Source: BMC Pregnancy Childbirth. 2006 Mar 19;6:8. doi: 10.1186/1471-2393-6-8 (PMC1468428; doi:10.1186/1471-2393-6-8)

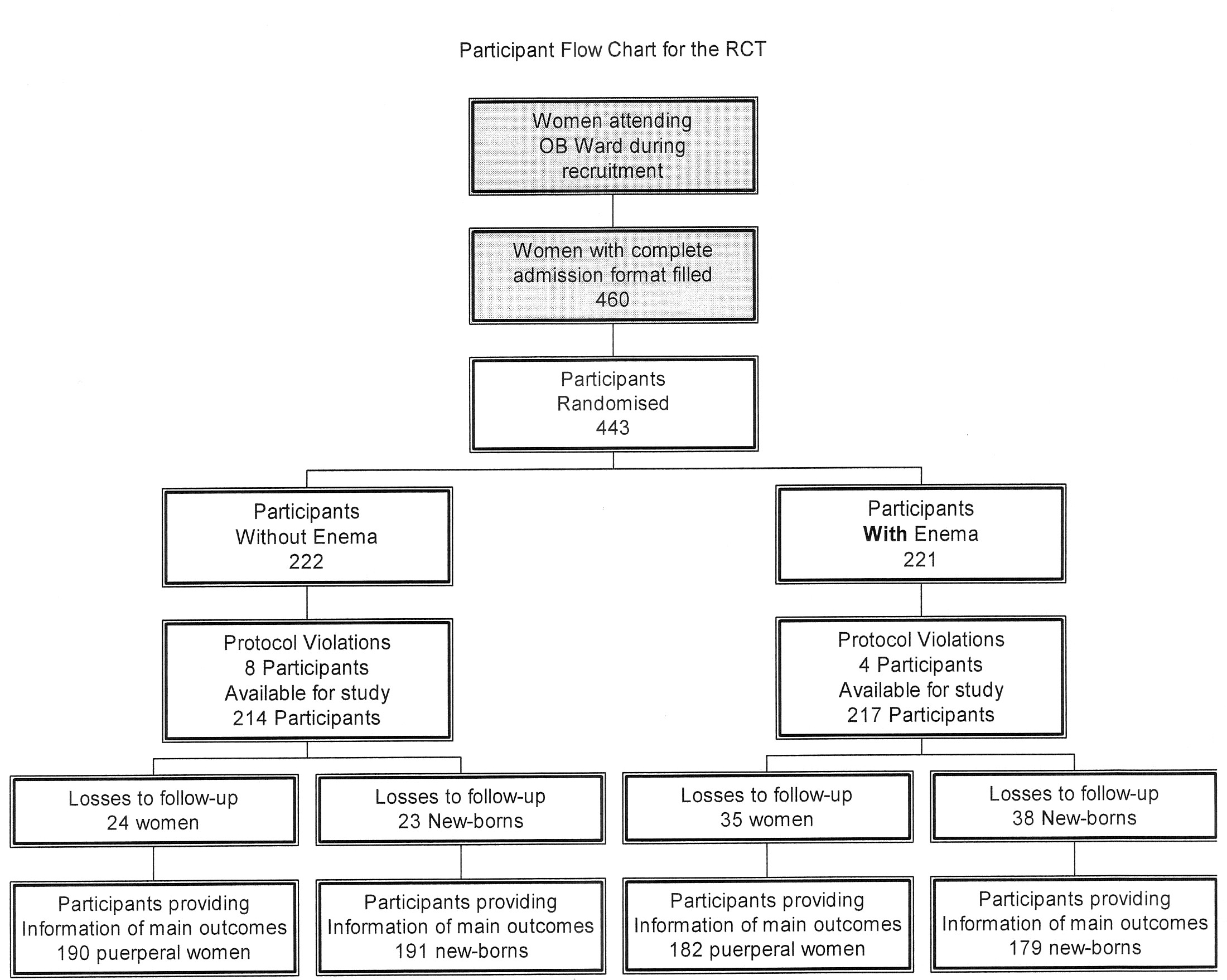

Supplement: Additional File 1 — Participant flow and follow-up. [file 1471-2393-6-8-S1.jpeg]
